# Convolutional neural networks based efficient approach for classification of lung diseases

**DOI:** 10.1007/s13755-019-0091-3

**Published:** 2019-12-23

**Authors:** Fatih Demir, Abdulkadir Sengur, Varun Bajaj

**Affiliations:** 1 Electrical and Electronics Engineering Dept., Technology Faculty, Firat University, Elazig, Turkey; 2 Discipline of ECE, IIITDM, Jabalpur, India

**Keywords:** Lung disease detection, Deep learning, Convolutional neural networks, Time-frequency images

## Abstract

Treatment of lung diseases, which are the third most common cause of death in the world, is of great importance in the medical field. Many studies using lung sounds recorded with stethoscope have been conducted in the literature in order to diagnose the lung diseases with artificial intelligence-compatible devices and to assist the experts in their diagnosis. In this paper, ICBHI 2017 database which includes different sample frequencies, noise and background sounds was used for the classification of lung sounds. The lung sound signals were initially converted to spectrogram images by using time–frequency method. The short time Fourier transform (STFT) method was considered as time–frequency transformation. Two deep learning based approaches were used for lung sound classification. In the first approach, a pre-trained deep convolutional neural networks (CNN) model was used for feature extraction and a support vector machine (SVM) classifier was used in classification of the lung sounds. In the second approach, the pre-trained deep CNN model was fine-tuned (transfer learning) via spectrogram images for lung sound classification. The accuracies of the proposed methods were tested by using the ten-fold cross validation. The accuracies for the first and second proposed methods were 65.5% and 63.09%, respectively. The obtained accuracies were then compared with some of the existing results and it was seen that obtained scores were better than the other results.

## Introduction

Respiratory system diseases affect people’s social, economic and health life significantly. For these reasons, a lot of researches are going on for early diagnosis and intervention in respiratory diseases. In this context, lung sound characteristics provide important clues in the diagnosis of respiratory abnormalities and infections. Auscultation is an effective technique in which physicians evaluate and diagnose the disease after using a stethoscope for lung disease. This method is both inexpensive and easy, and also it does not require internal intervention into the human body [1]. However, traditional stethoscopes may be exposed to external noise sounds, weaken the sound components above 120 Hz, and cannot filter the audio frequencies of the body in auscultation and cannot create permanent recordings in monitoring of the disease course [1]. In addition, accurate diagnosis of diseases requires highly experienced medical staff. Therefore, it is important to use electronic instrumentation and systems which operate with artificial intelligence and pattern recognition to assist doctors in decision making process. As a result, it is practically contributed to a specialist who works under stress, fatigue and intensive conditions.

### Related works

In [2], a data set consisting of crackle and non-crackle classes and a total of 6000 audio files were used for lung sound classification. Two feature extraction methods which use time–frequency (TF) and time-scale (TS) analysis were preferred for recognition of respiratory crackles. In the classification stage, k-Nearest Neighbors (k-NN), Support Vector Machine (SVM) and multi-layer sensor methods were used and the best accuracy was obtained with SVM classifier where the obtained accuracy score was 97.5%. In [3], two datasets namely continuous adventitious sound (CAS) and tracheal breath sound (TBS) were considered. TBS and CAS datasets were further divided into two sections: inspiratory and expiratory. TBS and CAS dataset have the following class labels; wheezing, stridor, rhonchi and mixture lung sounds. Distinction function, instantaneous kurtosis, and SampEn were used for feature extraction. The reported accuracy scores were in the range of 97.7% and 98.8% that were obtained with SVM classifier using the Radial Basis Function (RBF) kernel. In [4], MFCC was used for feature extraction of normal and wheeze sound files. Then, the method was trained and tested with the Gaussian Mixture Model (GMM), and the reported best accuracy was 94.2%. In [5], genetic algorithm and Fisher’s discriminant ratio were used to reduce dimension, and Higher Order Statistics (HOS) were used to extract features from respiratory sounds which consist of normal, coarse crackle, fine crackle, monophonic and polyphonic wheezes. The obtained accuracy score was 94.6%. In [6], the authors used the ICBHI 2017 challenge database which has normal, wheezes, crackles and wheezes plus crackles class labels. The ICBHI 2017 is a challenging database, since there are noises, background sounds and different sampling frequencies (4 kHz, 10 kHz, 44.1 kHz). In [7], spectral features and Decision Tree were chosen for feature extraction and classification, respectively. In [8], it was used MFCC at the stage of feature extraction, and was developed a method that uses Gaussian mixture models (GMM) and hidden Markov models (HMM) classifiers together at the stage of classification. In [5], authors chose short time Fourier transform (STFT) and STFT + Wavelet to extract features and principal component analysis (PCA) to reduce the process load while testing the algorithm performance with the SVM classifier.

In this paper, it was worked to boost the classification performance for ICBHI 2017 database which is quite challenging. In this context, spectrogram images were utilized to create time–frequency transformation from the lung sounds. These spectrogram images were used as input to the deep feature extraction and transfer learning. SVM and softmax classifiers were used for deep features and transfer learning approaches, respectively. The performances of proposed methods are evaluated by accuracy, sensitivity and specificity scores. The results were also compared with some of the existing results. The proposed schemes improved the classification performance of the lung sound discrimination.

## The methodology

**Fig. 1:**
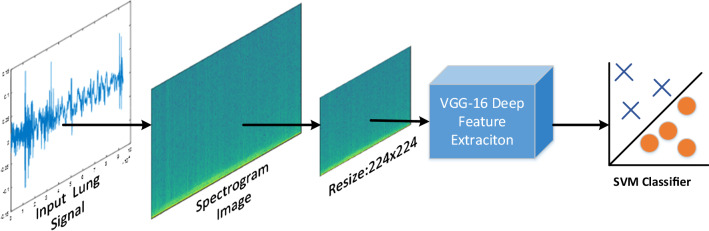
The proposed deep feature extraction and SVM classification methodology for lung sound classification

**Fig. 2:**
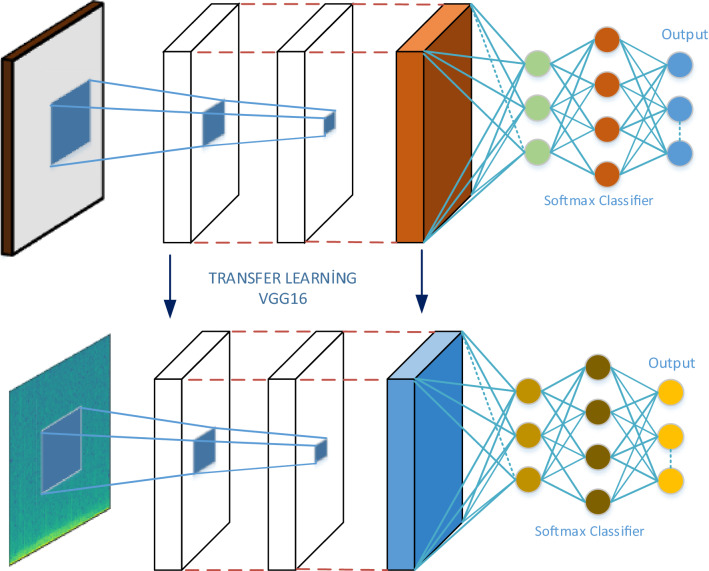
The proposed transfer learning methodology for lung sound classification

The proposed methods for lung sounds classification are shown in Figs. 1 and 2, respectively. The pre-trained VGG-16 model is considered for both deep feature extraction and fine-tuning. As seen in Figs. 1 and 2, the proposed methods initially convert the input lung sound signals into time–frequency images. The short time Fourier transform (STFT) is used for T-F image construction. Because lung sounds are recorded at different frequencies, the window sizes that should be used for the STFT are different. Window sizes are chosen between 0.01 and 0.025 times of the sampling frequency because they would better reveal the lung sound characteristics [14]. After the T-F images are constructed, they are resized to 224 × 224 (VGG16) because of being suitable with deep feature extraction and transfer learning.

The fully connected layers whose outputs are 4096-dimensional are used for deep feature extraction. Then, SVM algorithm was used to predict the all the class labels. The pre-trained VGG16 model was chosen for transfer learning in Fig. 2. The pre-trained VGG16 model is further trained with the input lung spectrogram images called fine-tuning. Since layers are configured for 1000 classes of ImageNet challenge, the last three layers of the VGG16 model are not taken into account in order to get fine-tuning adaptation.

### Spectrogram

The Short Time Fourier Transform (STFT) represents the frequency content of a (windowed) audio segment. Concatenating such representations over time allows for a waveform to be visualized as a 2-D function of time and frequency [15].

Considering a given signal x and the corresponding STFT representation F is calculated as:

1 $${\text{F}}\left( {{\text{n}},\upomega } \right) = \mathop \sum \limits_{{{\text{i}} = - \infty }}^{\infty } {\text{x}}\left( {\text{i}} \right)\upomega \left( {{\text{n}} - {\text{i}}} \right){\text{e}}^{{ - {\text{jwn}}}}$$

where ω(i) is a window function centered at the time n. The STFT representation provides some useful information about a waveform; e.g. what frequencies are present in a waveform and their strength. The temporal concatenation of the squared magnitude of windowed STFT representation, |F(n,ω)|^2^, is commonly referred to as the spectrogram.

### Deep approaches

#### Convolutional neural networks (CNN)

Convolutional neural networks (CNNs) take place as the category of deep neural networks, in terms of searching application to present categorization of images and analysis [10]. The categorization and attribute extraction is given by end-to-end learning architecture of CNNs. CNNs are composed of convolution, pooling and fully connected layers. The pooling and convolution layers are back-to-back utilized for the formation of network architecture and building high degree of discernable feature set for categorization. Categorization performing which use feature set obtained from previous layers is demanded for the fully connected layers. The traditional back propagation algorithm updates a great number of parameters in CNN model training.

The base purpose of the convolutional layer is to determine local connections of features from the previous layer and mapping their view to a feature map. For the filter $F\epsilon {\mathbb{R}}^{{2a_{1} + 2a_{2} }}$, the convolution operation of the input I with filter F is given in Eq. 2.

2 $$\left( {I*F} \right)_{n,m} = \mathop \sum \limits_{{k = - a_{1} }}^{{a_{1} }} \mathop \sum \limits_{{l = - a_{2} }}^{{a_{2} }} F_{k,l} I_{n - k,m - l}$$

where the filter *F* is $$\left[ {\begin{array}{*{20}c} {F_{{ - a_{1} - ,a_{2} }} } & \cdots & {F_{{ - a_{1} ,a_{2} }} } \\ \vdots & {F_{0,0} } & \vdots \\ {F_{{a_{1} , - a_{2} }} } & \cdots & {F_{{a_{1} ,a_{2} }} } \\ \end{array} } \right]$$.

A non-linear activation function such as ReLU $\left( {R\left( z \right) = \hbox{max} \left( {0,z} \right)} \right)$ is used to the feature map that is constituted by convolution operation. The aim of the max-pooling layer is to conjugate semantically convenient features came from the previous layer. The max-pooling layer executes down-sampling operation by splitting the previous layer into rectangular pooling regions, and calculating the maximum value of each region [19].

Fully connected layer, softmax classifier are existed in last stage of CNNs. The fully connected layer have a transmission mission between previous layer and classification layer. The fully connected layers can be summarized in three stages. In the first stage, the results of convolution and pooling layer are flattened and converted them to a column vector that will be an input layer for the latter stage. In the second stage, inputs from previous stage are taken for the feature analysis, and the weights are applied to predict the true labels. In the last stage, the final prediction scores for each class label is determined.

#### Transfer learning

In transfer learning (TL), which is contemporary trend in deep learning, the layers of a pre-trained network are shared or conveyed to other networks for fine-tuning or features extraction [11]. Initially, the training of a CNN model is carried out by a large dataset. After this process, training of pre-trained model is conducted once more by a smaller dataset to get fine tuning for developing estimated performance of CNN model. TL which provides satisfying tuning is more enduring than CNN model training from scratch. While initial layers represent features such as curves, color blobs, edges in CNN architecture, abstract and specific features are provided by final layers [16, 17].

#### Deep feature extraction

In deep feature extraction (DFE), which also performs on principle of transfer learning in place of training a pre-trained CNN model, the related feature vectors are extracted by using activation layers of CNN models [11]. While the previous layers’ activations present low-level image features such as edges, later or deeper layers present explicitly higher-level features for recognition of image. For instance, the activations of first and second fully connected layers provide feature representation in ImageNets.

### Classifiers

#### Support vector machine (SVM)

The SVM is an efficient classifier [12]. SVM aims to separate two classes by determining a hyperplane which maximizes the margin by optimization;

3 $$\begin{aligned} \mathop {\hbox{min} }\limits_{w,b,\xi } J\left( {\vec{w},\vec{\xi }} \right) = {\raise0.7ex\hbox{$1$} \!\mathord{\left/ {\vphantom {1 2}}\right.\kern-0pt} \!\lower0.7ex\hbox{$2$}}w^{T} w + C\mathop \sum \limits_{n = 1}^{N} \xi_{n} \\ st. & y_{n} \left[ {w^{T} \varphi \left( {x_{n} } \right) + b} \right] \ge 1 - \xi_{n} , \\ & \xi_{n} \ge 0, \;\;\;n = 1, \ldots ,N \\ \end{aligned}$$

where *w*, *b* and $\xi$ are the weight vector, bias and slack variable, respectively. $\varphi$ is known as the non-linear kernel function and *C* > 0 is a constant. In SVM procedure, the main goal is to find an optimal hyperplane, which minimizes the misclassification errors and maximize the margin size simultaneously. The most common way to deal with such problems is the use of Lagrange multipliers to transfer the problem from the primal space to a dual space. Introducing *n* nonnegative Lagrange multipliers α_1_, α_2_,…, α_n_ ≥ 0 associated with the inequality constraints defined in Eq. 4 results in Eq. 5:

4 $$L\left( a \right) = \mathop \sum \limits_{i = 1}^{n} \upalpha_{i} - \frac{1}{2} \mathop \sum \limits_{i = 1}^{n} \mathop \sum \limits_{j = 1}^{n} \upalpha_{i} \upalpha_{j} y_{i} {\text{y}}_{j} {\text{x}}_{i} \cdot {\text{x}}_{j}$$

Subject to:

5 $$\mathop \sum \limits_{j = 1}^{n} \upalpha_{i} y_{i} = 0, \;\;\; 0 \le \upalpha_{i} \le c, \;\;\;i = 1,2, \ldots ,n$$

To address non-linearity, data can be mapped to a higher dimensional space created using a mathematical projection and known as the kernel trick. Because in this optimization problem, only the dot product of two vectors appears in the feature space, by replacing *x* with its mapping in the feature space, the kernel function *k* can be defined as $k(x_{i} x_{j} ) = \varPhi (x_{i} )\varPhi (x_{j} )$. Using a kernel function, the optimization function accounts to maximizing Eq. 6.

6 $$L\left( a \right) = \mathop \sum \limits_{i = 1}^{n} \upalpha_{i} - \frac{1}{2} \mathop \sum \limits_{i = 1}^{n} \mathop \sum \limits_{j = 1}^{n} \upalpha_{i} \upalpha_{j} y_{i} {\text{y}}_{j} k({\text{x}}_{i} \cdot {\text{x}}_{j} )$$

#### Softmax classifier

The generalized binary form of logistics regression is used for the Softmax classifier. Similar to hinge loss functions, *g* which is mapping function is the linear dot product of $x_{i}$ symbolized as input data and of $\omega$ symbolized as weight matrix, as shown in Eq. 7 [13].

7 $$g\left( {x_{i} ,\omega } \right) = \omega \cdot x_{i}$$

Yet, unlike hinge loss functions, results are interpreted as non-normalized log possibilities for class tags. Therefore, hinge loss function with cross entropy loss function is changed, and the loss function is ultimately as shown in Eq. 8.

8 $$F_{i} = - \log \left( {\frac{{e^{{t_{{y_{i} }} }} }}{{\mathop \sum \nolimits_{j} e^{{t_{j} }} }}} \right), \;\;\; t = g\left( {x_{i} ,\omega } \right)$$

## Experimental works

### Database

ICBHI 2017 Challenge Dataset consists of 920 audio files. According to class labels, each one of these audio files is divided to cycles. The labelling details of a sample lung sound is given in Table 1. The 20-s audio file in Table 1 is divided into 9 cycles according to the start and end times. The number of these cycles and the sampling frequency (4 kHz, 10 kHz, 44.1 kHz) are different for each audio file. The class labels of the divided cycles are set by checking the values of wheezes and crackles columns in Table 1. The class labels are crackles, wheezes, normal and wheezes plus crackles if the wheezes and crackles values are 1-0, 0-1, 0-0 and 1-1. Also the total number of ICBHI 2017 dataset cycles is shown to class labels in Table 2.

**Table 1:** Cycle info for an audio file of ICBHI 2017 database

Cycles	Start time	End time	Crackles	Wheezes
1	0.804	3.256	0	0
2	3.256	5.566	0	0
3	5.566	7.851	0	1
4	7.851	10.054	0	1
5	10.054	12.066	1	0
6	12.066	14.47	1	0
7	14.47	16.696	1	1
8	16.696	18.887	1	1
9	18.887	19.792	1	1

**Table 2:** The total number of ICBHI 2017 dataset cycles

Dataset	Total
Number of cycles with crackles	1864
Number of cycles with wheezes	886
Number of cycles with both	506
Number of normal cycles	3642
Number total of cycles	6898

### Results

**Fig. 3:**
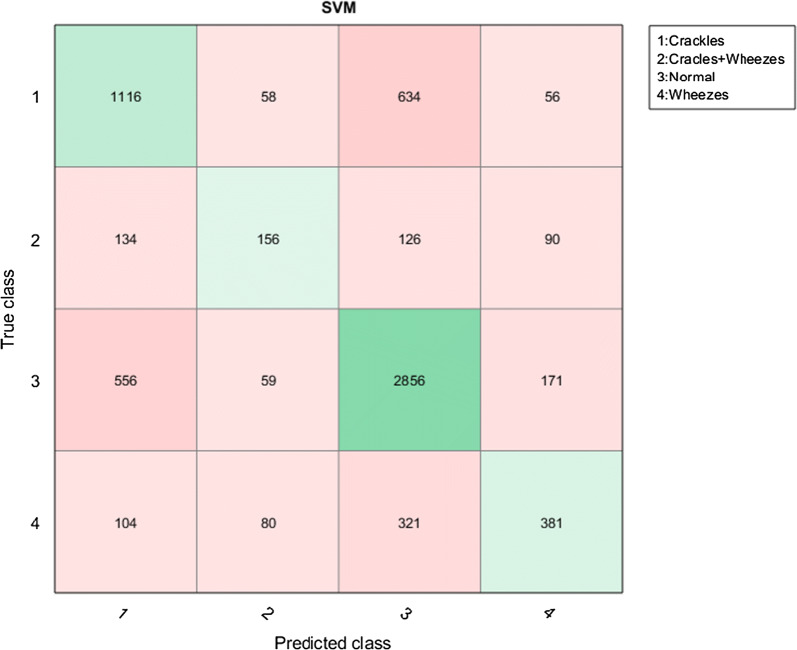
Confusion matrix for lung sound classification

**Fig. 4:**
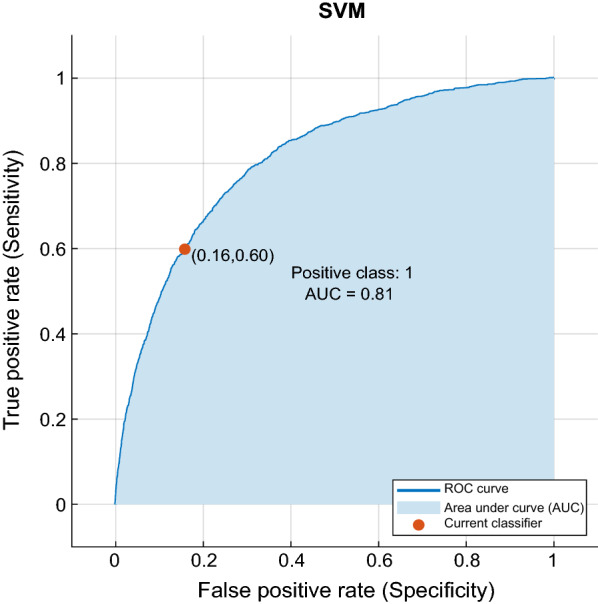
ROC curve for lung sound classification

All coding was conducted on Matlab using a computer having an Intel Core i7-4810 CPU and 32 GB memory. For spectrogram creation, we used Hamming window of with 1024 ms and the number of the FFT was chosen as 3000. The window-overlap sizes were selected as 512-64 for 44.1 kHz sampling frequency, 128-16 for 10 kHz sampling frequency and 64-8 for 4 kHz sampling frequency. In the first proposed method, 4096 features were extracted by using VGG-16 Model for each audio file over the fc6 fully connected layer. Matlab Classification Learner Tool (MCLT) was used in classification stage of the work. The testing process is carried out by using ten-fold cross validation test. The SVM parameters were automatically assigned by the MCLT. The obtained best accuracy was 65.5% by using cubic SVM classifiers. In the second method, 50% dropout was applied to prevent over-fitting in the final layer of the network used as fine-tuning of transfer learning. The obtained best accuracy by using fine-tuning of VGG-16 CNN model with softmax classifier was 63.09%. In Fig. 3, confusion matrix which is a performance measurement for classification was shown for four classes. It has been shown different combinations for all the classes in the confusion matrix, which it is the numbers of true positive, false positive, true negative and false negative. For example, the numbers of true positive, false positive, true negative and false negative were 1116, 748, 3393 and 794 for the crackles class, respectively. In Fig. 4, the true positive rate (Sensitivity) was drawn in function of the false positive rate (Specificity) for different cut-off points. This drawing is called Receiver Operating Characteristic (ROC) curve. Formulas of sensitivity, specificity, false alarm rate, precision and F-score are given in Eqs. 9–13 respectively.

9 $$Sensitiviy = \frac{True \;Positive}{True\;Positive + False\;Negative}$$

10 $$Specificity = \frac{True\;Negative}{True\;Negative + False\;Positive}$$

11 $$False\;Alarm \;Rate = 1 - specificity$$

12 $$Precision = \frac{True\;Positive}{True\;Positive + False \;Positive}$$

13 $$F - score = 2*\frac{Precision \times Sensitiviy}{Precision + Sensitiviy}$$

Table 3 shows the classification accuracies of the proposed methods and other methods using the ICBHI 2017 data set. In the lines of the methodology column of Table 3, the methods before the comma represent the feature extractions, and the methods after the comma represent the classifiers. In addition, the classification accuracies together with ResNet-50 and AlexNet CNN models are shown for both proposed methods in Table 4.

**Table 3:** The classification accuracies of the methods using ICBHI 2017 Database

Authors	Methodology	Accuracy (%)
Jakovljević et al. [8]	MFCC, Hidden Markov model	39.56
Chambres et al. [7]	Low level feature, decision tree	49.62
Serbes et al. [9]	STFT + Wavelet, SVM classifier	57.88
The first proposed method	Deep Feature with CNN model, and SVM classifier	**65.50**
The second proposed method	Transfer learning with CNN Model, and softmax classifier	63.09

The best score is given in bold

**Table 4:** The classification accuracies together with AlexNet and ResNet-50 CNN models

CNN models	Deep feature + SVM (Acc %)	Transfer learning +Softmax (Acc %)
AlexNet	60.5	61.23
ResNet-50	59.10	60.05
VGG-16	**65.50**	63.09

The best score is given in bold

As shown Table 4, the best classification performance among the CNN models was achieved with the VGG-16 CNN model in both proposed methods. For both the class and average of the classes, the other evaluation criterias including sensitivity, specificity, false alarm rate, precision and *F*-*score* are given in Table 5.

**Table 5:** The other evaluation criterias for the proposed method

	Sensitivity	Specificity	False alarm rate	Precision	*F*-score
Crackles label	0.60	0.81	0.19	0.58	0.59
Crackles + wheezes label	0.31	**0.96**	**0.04**	0.44	0.36
Normal label	**0.78**	0.60	0.40	**0.73**	**0.75**
Wheezes label	0.43	0.93	0.07	0.55	0.48
Average values	0.53	0.83	0.17	0.57	0.55

The best scores are given in bold

## Conclusions

This work focuses on the automatic diagnosis of lung diseases which is one of the most important issues in public health. There have been many studies on this subject in the literature, but no challenging data sets including background sounds, noises and different sampling frequencies have been used for lung sound classification. Also most of the work consists of traditional methods. In recognition problem of lung sounds, deep learning, which is state of the art method, is handled to boost the classification performance. In the pre-processing stage of the proposed methods, images that meet one-to-one spectrogram properties were obtained with colormap to extract deep feature and apply fine-tuning. In both deep learning methods, the VGG-16 model of CNN was used to perform feature extraction. In addition, AlexNet and ResNet-50 models of CNN were given classification performance, and VGG-16 model was preferred for the proposed methods because it gave better classification accuracy. The classification accuracies for both proposed methods have been significantly improved for ICBHI 2017 Database containing lung sounds that are difficult to classify. According to other published methods, the classification accuracy was boosted by 7.62% with first proposed method which use deep feature extraction and SVM classifier and by 5.18% with the second proposed method which use transfer learning and softmax classifier.
